# Identification of Genes Related to Beak Deformity of Chickens Using Digital Gene Expression Profiling

**DOI:** 10.1371/journal.pone.0107050

**Published:** 2014-09-08

**Authors:** Hao Bai, Jing Zhu, Yanyan Sun, Ranran Liu, Nian Liu, Dongli Li, Jie Wen, Jilan Chen

**Affiliations:** Key Laboratory of Genetics Resources and Utilization of Livestock, Institute of Animal Science, Chinese Academy of Agricultural Sciences, Beijing, China; Wageningen UR Livestock Research, Netherlands

## Abstract

Frequencies of up to 3% of beak deformity (normally a crossed beak) occur in some indigenous chickens in China, such as and Beijing-You. Chickens with deformed beaks have reduced feed intake, growth rate, and abnormal behaviors. Beak deformity represents an economic as well as an animal welfare problem in the poultry industry. Because the genetic basis of beak deformity remains incompletely understood, the present study sought to identify important genes and metabolic pathways involved in this phenotype. Digital gene expression analysis was performed on deformed and normal beaks collected from Beijing-You chickens to detect global gene expression differences. A total of >11 million cDNA tags were sequenced, and 5,864,499 and 5,648,877 clean tags were obtained in the libraries of deformed and normal beaks, respectively. In total, 1,156 differentially expressed genes (DEG) were identified in the deformed beak with 409 being up-regulated and 747 down-regulated in the deformed beaks. qRT-PCR using eight genes was performed to verify the results of DGE profiling. Gene ontology (GO) analysis highlighted that genes of the keratin family on GGA25 were abundant among the DEGs. Pathway analysis showed that many DEGs were linked to the biosynthesis of unsaturated fatty acids and glycerolipid metabolism. Combining the analyses, 11 genes (*MUC*, *LOC426217*, *BMP4*, *ACAA1*, *LPL*, *ALDH7A1*, *GLA*, *RETSAT*, *SDR16C5*, *WWOX*, and *MOGAT1*) were highlighted as potential candidate genes for beak deformity in chickens. Some of these genes have been identified previously, while others have unknown function with respect to thus phenotype. To the best of our knowledge, this is the first genome-wide study to investigate the transcriptome differences in the deformed and normal beaks of chickens. The DEGs identified here are worthy of further functional characterization.

## Introduction

**Figure 1:**
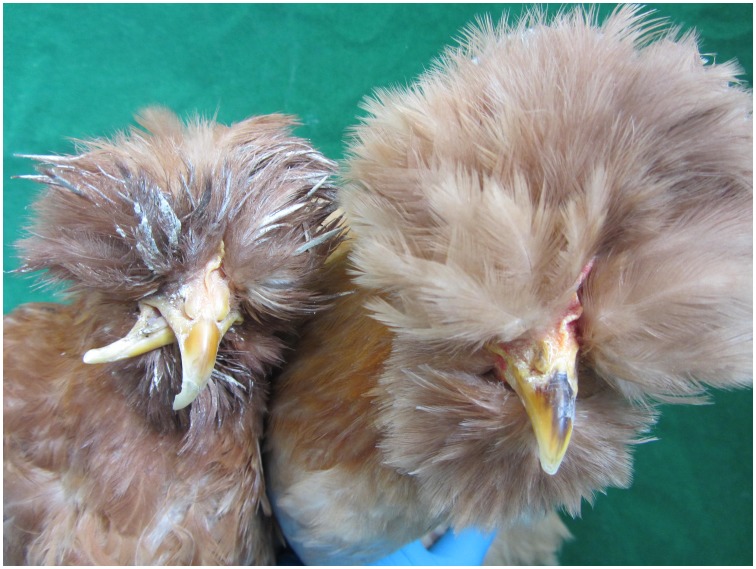
The deformed (left) and normal (right) beaks of Beijing-You chickens. The chicken with a deformed beak had problem with feeding, drinking, and preening and therefore showed lower body weight and poor mental condition.

The beak is an external structure of birds consisting of the upper and lower mandibles covered with a thin keratinized layer of epidermis [1]. It is used for many important activities such as feeding, drinking, fighting, and preening. In addition to striking morphological differences between species, beak deformities of different forms (noticeably elongated, crossed, bent at right angles and so on) have been documented in many wild birds including Japanese quails [2], Brown-headed Cowbird [3], Black Capped Chickadees [4], Northwestern Crows [5], African Seedcracker [6], and Senegal Parrots [7]. Frequencies of up to 3% beak deformity (Figure 1) have been found in some indigenous breeds, such as Beijing-You chickens, the breed studied here [8] and Qingyuanma chickens. Chickens with deformed beaks have reduced feed intake, growth rate, and impaired normal behaviors like preening and social contact with their mates. Beak deformity, therefore, represents an economic as well as an animal welfare problem in the poultry industry.

Intensive researches have been performed to seek the pinpoint causes of the malformation in birds, especially the wild birds. Diseases, environmental contaminants, and lack of nutriments are some likely contributing factors [9], [10]. Further complexity stems from the very early embryonic determination of rostral development likely influencing subsequent growth, which continues long after hatching [11]. The underlying pathology and possible mechanisms of the disorder, however, remain incompletely understood and have baffled scientists for a number of years.

According to our observations in a pedigreed Beijing-You chickens population, in the absence of known environmental factors contributing to the malformation, birds with deformed beaks appear consistently in each generation and cannot be eliminated from a population simply on the basis of the phenotype. This indicated the genetic effects for the formation of beak deformity. Previously recognized genetic factors associated with beak deformity include the reported candidate genes such as fibroblast growth factor 8 (*FGF8*) [12] and bone morphogenetic protein 4 (*BMP4*) [13]. The over-expression of homeobox A1 (*HOXA1*) and homeobox D3 (*HOXD3*) may result in beak deformity in chicks [14]. The approaches used in these existing studies, while mechanistically rigorous, have the limitation of lower-throughput for comprehensive screening and critical underlying genes can be overlooked.

Based on the new generation of high-throughput sequencing and high-performance computing technology, digital gene expression (DGE) can be used to capture the entire transcriptome of a given tissue. The aim of this first study was to identify genes related to the beak deformity phenotype using DGE as a starting point for revealing the molecular genetic mechanisms underlying the condition. The results identified sets of up-regulated and down-regulated genes in the deformed beak compared to normal. Combined with the follow-up gene ontology and pathway enrichment analyses of the differentially expressed genes (DEGs), some candidate genes and pathways related to beak deformity are proposed and discussed.

## Materials and Methods

### Samples and RNA isolation

The lower mandibles of the beaks were collected from 6 Beijing-You cocks of 56 days of age: 2 with crossed beaks (individuals 1 and 2) and 4 with normal beaks (individuals 3, 4, 5, and 6). Individuals 1, 3, 5, and 6 were full sibs; as were individuals 2 and 4. The chickens had been incubated and housed under the same conditions. Total RNA of the lower mandibles of the beaks was isolated using TRIzol (Invitrogen, USA) according to the manufacturer's instructions followed by RNase-free DNase treatment (TIANGEN, China). The quality and quantity of the total RNA was assessed with a Biophotometer Plus (Eppendorf, Germany). RNA from the beaks of individual 1 (deformed) and 6 (control) was used to determine the DEGs at the genome-wide level using DGE profiling. Four RNA sample pairs (2 and 4, 1 and 3, 1 and 5, and 1 and 6) were used to determine relative abundance of 8 transcripts using quantitative real-time PCR (qRT-PCR).

### DGE-tag profiling and sequence annotation

cDNA was prepared from RNA1 and 6, digested and ligated with Illumina adaptors, as recommended, to generate 17 bp tagged fragments for Solexa sequencing, which was performed by BGI (Beijing, China).

Clean-tags were obtained by filtering the adaptor sequences and removing low-quality sequences (those with some ambiguous bases) then mapped to the reference genome and genes of chickens (*Gallus_gallus-4.0*) available at http://www.ncbi.nlm.nih.gov. Only tags with a perfect match or 1 mismatch were further considered and annotated. The expression level of each gene was estimated by the frequency of clean tags and then normalized as TPM (number of transcripts per million clean tags) [15], [16].

### Identification of DEGs

The differential expression of genes between the deformed and normal beaks was determined with a rigorous algorithm method, which has been developed to identify DEGs between two samples by the BGI (Beijing, China), referring to the methods published by Audic and Claverie [17]. Denote the number of unambiguous clean tag from gene A as x, as every gene's expression occupies only a small part of the library, the p(x) is in the Poisson distribution:



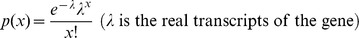



The total clean tag number of the sample 1 is N1, and total clean tag number of sample 2 is N2; gene A holds x tags in sample1 and y tags in sample 2. The probability of gene A expressed equally between two samples can be calculated with:



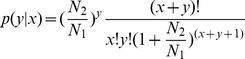



*P*-Value corresponds to differential gene expression test. Genes were deemed as being significantly differentially expressed with a *P*-value <0.005, FDR (false discovery rate) <0.001, and a 2-fold relative change threshold (|log2-Ratio (deformed beak/normal beak)| >1) [18] in the sequence counts across the 2 libraries. All the genes in the present study were referenced from the gene dataset of GenBank.

### Gene ontology (GO) and pathway enrichment analysis of DEGs

Based on the Gene Ontology Database (http://www.geneontology.org/) [19] and the KEGG pathway (http://www.genome.jp/kegg/) [20], GO functional enrichment analysis and pathway enrichment analysis were used to identify significantly enriched functional classification and metabolic pathways in the DEGs, respectively [21]. The formula was [22]:



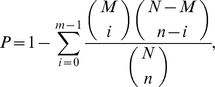
where N is the total number of genes with GO/KEGG functional annotations, and n is the number of DEGs in N, M is number of the genes with specific GO/KEGG annotations, and m is the number of DEGs in M. In this study, *P*-value ≤0.05 indicated significantly enriched GO terms and pathways.

### qRT-PCR analysis for validating the DEGs

Eight genes (Table 1), selected from the DEGs, were analyzed by qRT-PCR with an ABI 7500 Real-time Detection System (Applied Biosystems, USA) to validate the DGE results. The total RNA samples (1 µg) of four comparison pairs were used. The housekeeping gene *β*-actin was used as the endogenous control. Gene-specific primers were designed from the reference Unigene sequences available in GenBank using Primer Premier 5.0 (Table 1). qRT-PCR was performed in triplicate according to manufacturer specifications (TaKaRa SYBR PrimeScript RT-PCR Kit, Dalian, China). After denaturing at 95°C for 30 s, 40 cycles of 95°C for 5 s and 60°C for 32 s were used, followed by thermal denaturing to generate melting curves to verify amplification specificity. The comparative CT method (2^−ΔΔCT^) was used to determine the fold-changes in transcript abundance. Student's *t*-tests were used to evaluate the differences between the RNA samples of deformed and normal beaks.

**Table 1:** Gene-specific primers used in qRT-PCR.

Gene^a^	GenBank No.	Primer Sequence	Tm (°C)	Product length (bp)
*NPM3*	XM_001233763	F: 5'-CCATCGTGCCTGCCAAGAAG-3'	60	132
		R: 5'-AAAAAAGGCAGCAAAAGTTG-3'		
*LPL*	NM_205282	F: 5'-GGTAGACCAGCCATTCCTGA-3'	60	192
		R: 5'-CACCAGTCTGACCAGCTGAA-3'		
*BMP4*	NM_205237	F: 5'-AGTCCGGAGAAGAGGAGGAG-3'	60	164
		R: 5'-GCTGAGGTTGAAGACGAAGC-3'		
*SGOL1*	NM_001199648	F: 5'-ACAGCGTGAGCAGAAACTCC-3'	60	159
		R: 5'-GGTGCGTTCCCTTTGCTTAG-3'		
*KRT19*	NM_205009	F: 5'-AAGATCCTGGCCGATATGAG-3'	60	122
		R: 5'-TCGGTATTGACGGCTAACTC-3'		
*sKer*	XM_428869	F: 5'-GCTATGGAGGATCTCAGGGT-3'	60	138
		R: 5'-AGAGGCCAGAGCTGTAGGAC-3'		
*MMP7*	NM_001006278	F: 5'-GGAAGAGGTGGCACATTAGC-3'	60	167
		R: 5'-ACATTTGAGTGGGCGAGTCC-3'		
*MUC*	XM_421033	F: 5'-GCAACGGCATCAATGACTTC-3'	60	168
		R: 5'-TGCTACACTGCTTCTCTGAC-3'		
*β-actin*	NM_205518	F: 5′-GAGAAATTGTGCGTGACATCA-3′	60	152
		R: 5′-CCTGAACCTCTCATTGCCA-3′		

a *NPM3*  =  nucleophosmin/nucleoplasmin 3; *LPL*  =  lipoprotein lipase; *BMP4*  =  bone morphogenetic protein 4; *SGOL1*  =  shugoshin-like 1; *KRT19*  =  Keratin 19; *sKer*  =  similar to Scale keratin; *MMP7*  =  matrix metalloproteinase 7; *MUC*  =  mucin protein.

### Ethics statement

The Institutional Animal Care and Use Committee at Institute of Animal Science, Chinese Academy of Agricultural Sciences approved all procedures involving the use of animals. All efforts were made to minimize the suffering of animals.

## Results

### Analysis of DGE libraries and tag mapping

The total number of sequenced tags (6,027,921 for RNA1 (deformed beak) (Sequence Read Archive accession number: SRR1514179) and 5,807,305 for RNA6 (normal beak) (Sequence Read Archive accession number: SRR1514178)) were obtained from the Solexa sequencing. After filtering the adaptor sequences and removing the low-quality tags, 5,864,499 and 5,648,877 clean tags were retained (Table 2). Considering the robustness of subsequent data analysis, only tags with more than 1 copy were considered for further analysis.

**Table 2:** Statistics of tag mapping against reference gene and genome sequence of the chicken.

	Deformed beak		Normal beak	
	Total number	Distinct tag number	Total number	Distinct tag number
Raw data	6,027,921	262,179	5,807,305	251,861
Clean tags	5,864,499	106,180	5,648,877	100,893
All tags mapping to genes	2,935,689	41,530	2,925,791	42,794
Unambiguous tag mapping to genes	2,566,707	38,420	2,452,094	39,666
All tag-mapped genes	10,425	10,425	10,725	10,725
Unambiguous tag-mapped genes	9,096	9,096	9,328	9,328
Mapping to genome	1,444,399	38,761	1,275,093	36,268
Unknown tags	1,484,411	25,889	1,447,993	21,831

A reference gene database that included 19,131 Unigene sequences was preprocessed for tag mapping. As shown in Table 3, genes containing a CATG motif, accounted for 92.28% of all reference genes. Unambiguous tags accounted for 95.15% of the total reference tags. The data indicated that a high proportion of the entire chicken genome was expressed in the beak samples.

**Table 3:** The number of total reference genes and tags.

	Number	Percentage
Reference gene	19,131	
Genes with CATG site	17,655	92.28%
Total reference tags	153,121	
Unambiguous Tags	145,689	95.15%
Ambiguous tags	7,432	4.85%

**Figure 2:**
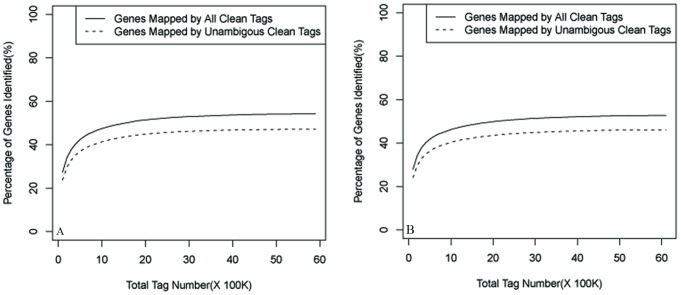
Sequencing saturation analysis for RNA from the deformed (A) and normal beak (B).

Saturation analysis of the sequencing data showed that the genes that were mapped by all clean tags and unambiguous clean tags were saturated when the tag counts approached 2 million (Figure 2). Therefore, the sequencing depth used here was sufficient for the transcriptome coverage.

### Comparison and analysis of DEGs

**Figure 3:**
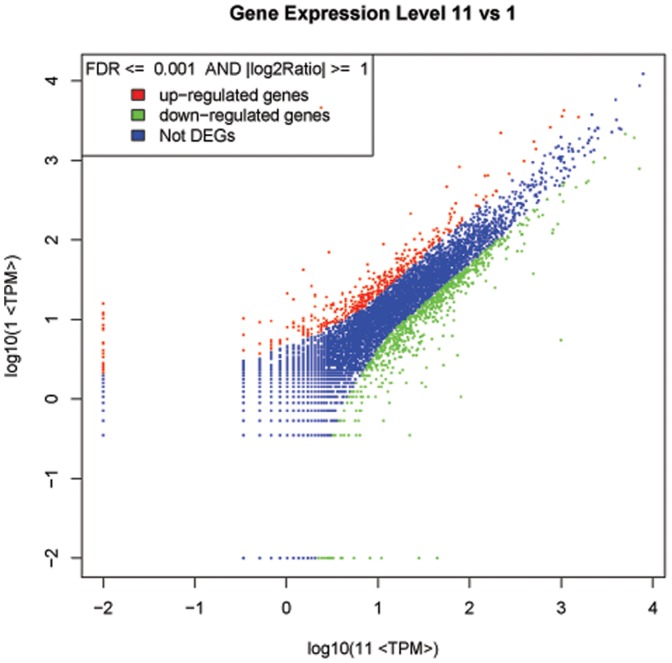
Comparison of gene expression between libraries of deformed and normal beak RNA. Blue dots represent the transcripts with no significant differential expression. Red and green dots represent transcripts more abundant in the test sample and control, respectively. FDR <0.001 and |log2-Ratio| ≧ 1 were used as the thresholds to judge the significance of gene expression difference.

**Figure 4:**
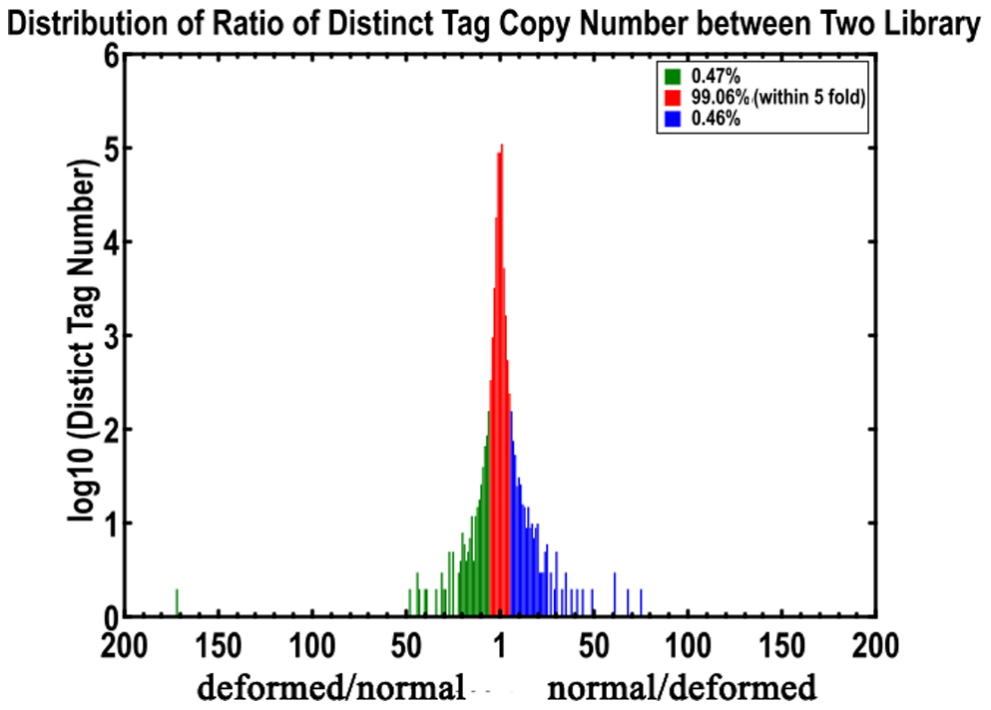
Distribution of ratios of distinct tag copy number between libraries of deformed and normal beak RNA. Red region represents the differentially expressed tags with differential expression less than 5-fold. Blue and green region represent the up- and down-regulated tags of more than 5-fold, respectively.

The transcripts detected with at least 2-fold differences (FDR <0.001 and |log2-Ratio (deformed beak/normal beak)| ≧ 1) in the deformed-beak library compared to the normal-beak library are shown in Figure 3. Most (99.06%) of the ratios of distinct tag copy number between the 2 libraries were within 5-fold (Figure 4). From these 2 libraries, 1,156 significant DEGs were identified, of which 409 (35.4%) were higher and 747 (64.6%) were lower in the deformed beak. A number of transcripts with extreme differences in abundance (|log2-Ratio (deformed beak/normal beak)| ≧ 9) are shown in Table 4. The detailed lists of DEGs are provided in Table S1 (up-regulated) and Table S2 (down-regulated). Based on the differential ratios, claw keratin-like (*LOC426217*) (|log2-Ratio (deformed beak/normal beak)|  = 10.91) and mucin protein (*MUC*) (|log2-Ratio (deformed beak/normal beak)|  = −12.11) are the most up- and down-regulated genes, respectively. They can be denoted as being likely important candidate genes related to beak deformity.

**Table 4:** Some extremely differentially expressed genes (|log2-Ratio (deformed beak/normal beak)| ≧ 9).

Species	Gene^a^	log2-Ratio(deformed/normal)
Gallus gallus	*LOC426217*	10.91
Gallus gallus	*NPM3*	10.62
Gallus gallus	*LPL*	10.25
Gallus gallus	*RBP7*	10.17
Gallus gallus	*NUBP2*	10.00
Gallus gallus	*ARL6IP1*	9.73
Gallus gallus	*ABF1*	9.61
Gallus gallus	*RNG213*	9.54
Gallus gallus	*THOC3*	9.00
Gallus gallus	*DGCR14*	−9.09
Gallus gallus	*C3orf38*	−9.09
Gallus gallus	*KRT19*	−9.68
Gallus gallus	*SGOL1*	−9.68
Gallus gallus	*sKer*	−10.09
Gallus gallus	*NMP7*	−11.45
Gallus gallus	*MUC*	−12.11

a *LOC426217*  =  claw keratin-like; *NPM3*  =  nucleophosmin/nucleoplasmin 3; *LPL*  =  lipoprotein lipase; *RBP7*  =  retinol binding protein 7 cellular; *NUBP2*  =  nucleotide binding protein 2 (MinD homolog *E. coli*); *ARL6IP1*  =  ADP-ribosylation factor-like 6 interacting protein 1; *ABF1  =  activated B-cell factor 1*; *RNG213*  =  ring finger protein 213; *THOC3*  =  THO complex 3; *DGCR14*  =  DiGeorge syndrome critical region gene 14; *C3orf38*  =  chromosome 3 open reading frame 38; *KRT19*  =  Keratin 19; *SGOL1*  =  shugoshin-like 1; *sKer*  =  similar to Scale keratin; *MMP7*  =  matrix metalloproteinase 7; *MUC* =  mucin protein.

### GO enrichment analysis of DEGs

**Figure 5:**
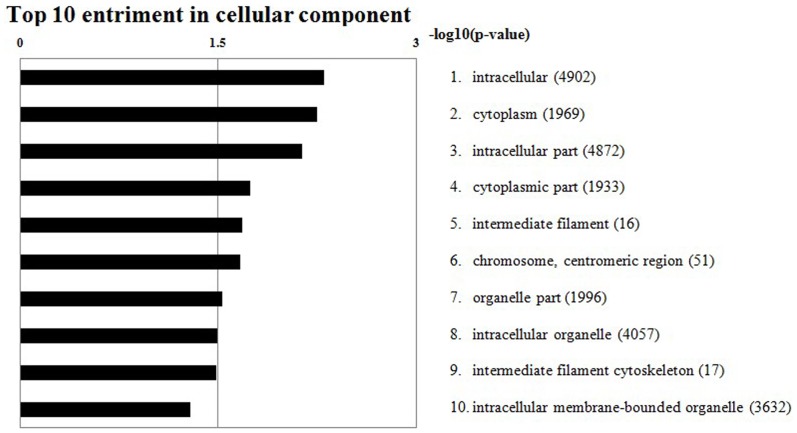
Top 10 enrichment cellular components. The numbers of genes involved in the components are indicated in parentheses.

The ontology database covers 3 domains: cellular component, molecular function, and biological process. When GO analysis results of the presently identified DEGs were examined, there were no significantly enriched GO terms on the ontology of molecular function or biological process. The DEGs were significantly enriched, however, on 9 terms of the ontology of cellular component category (Figure 5): GO0005622 intracellular (426 genes), GO0005737 cytoplasm (197 genes), GO0044424 intracellular part (423 genes), GO0044444 cytoplasmic part (191 genes), GO0005882 intermediate filament (7 genes), GO0000775 chromosome, centromeric region (13 genes), GO0044422 organelle part (195 genes), GO0043229 intracellular organelle (359 genes), and GO0045111 intermediate filament cytoskeleton (7 genes). These DEGs, therefore, were mainly associated with intracellular, cell organelle, and cytoskeletal terms, where they play their roles.

### Pathway enrichment analysis of DEGs

**Figure 6:**
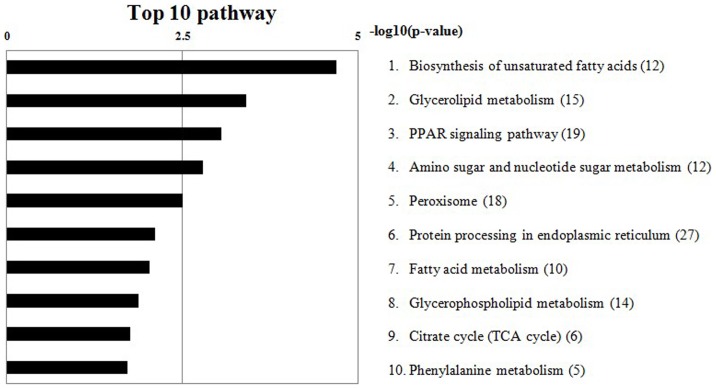
Top 10 enrichment pathways. The numbers of genes involved in the pathways are indicated in parentheses.

Pathway functional significant enrichment analysis showed that some DEGs were associated with biochemical metabolism and signal transduction pathways. All the DEGs were involved in 210 pathways and the top 10 enrichment pathways of the DEGs are shown in Figure 6. The results revealed that, *LOC426217* was not involved in any pathways. *MUC* was from salivary secretion pathway. *BMP4* was involved in the pathways of cancer, basal cell carcinoma, hedgehog signaling, TGF-beta signaling and cytokine-cytokine receptor interaction. Significantly enriched pathways included biosynthesis of unsaturated fatty acid (KO 01040 with 12 genes) and glycerolipid metabolism (KO 00561 with 14 genes) (Table 5). The details of the two pathways are shown in Figures S1 and S2, respectively. Four DEGs were involved in the two pathways viz. acetyl-CoA acyltransferase 1 (*ACAA1*), Lipoprotein Lipase (*LPL*), Aldehyde Dehydrogenase 7 family member A1 (*ALDH7A1*), and Alpha-galactosidase A (*GLA*). Although not detected as significantly enriched, retinol acid metabolism pathway was highlighted as 4 DEGs viz. Retinol saturase (*RETSAT*), short chain dehydrogenase/reductase family 16C, member 5 (*SDR16C5*), WW domain containing oxidoreductase (*WWOX*), and Monoacylglycerol O-acyltransferase 1 (*MOGAT1*) were identified to be involved.

**Table 5:** The two most significantly enriched pathways and the involved differentially expressed genes (DEG).

Biosynthesis of unsaturated fatty acids		Glycerolipid metabolism	
DEGs^a^	log2-Ratio (deformed/normal)	DEGs	log2-Ratio (deformed/normal)
*SCD5*	1.41	*LPL*	10.25
*GLG1*	−1.01	*PNPLA2*	8.54
*ELOVL6*	−1.08	*PPAP2A*	7.96
*FAD*	−1.19	*PPAP2B*	2.06
*PTPLB*	−1.20	*AGPAT6*	1.46
*ACAA1*	−1.35	*SHROOM3*	1.18
*PECR*	−1.43	*ALDH7A1*	−1.06
*MFSD4*	−1.54	*AKR1B10*	−1.14
*DLEC1*	−1.87	*DGKD*	−1.20
*PTPLAD1*	−1.92	*GK5*	−1.23
*HSDL1*	−2.31	*GLA*	−1.26
*LOC423119*	−3.37	*MOGAT1*	−1.28
		*AR*	−3.01
		*LIPG*	−3.01

a *SCD5*  =  stearoyl-CoA desaturase 5; *GLG1*  =  golgi glycoprotein 1; *ELOVL6*  =  ELOVL family member 6; *PTPLB*  =  protein tyrosine phosphatase-like B; *ACAA1*  =  acetyl-CoA acyltransferase 1; *PECR*  =  privacy and electronic communications regulations; *MFSD4*  =  major facilitator superfamily domain containing 4; *DLEC1*  =  deleted in lung and esophageal cancer 1; *PTPLAD1*  =  protein tyrosine phosphatase-like A domain containing 1; *HSDL1*  =  hydroxysteroid dehydrogenase like gene; *LOC423119*  =  Gallus gallus fatty acid desaturase 1-like; *LPL*  =  lipoprotein lipase; *PNPLA2*  =  patatin-like phospholipase domain containing 2; *PPAP2A*  =  phosphatidic acid phosphatase 2A; *PPAP2B*  =  phosphatidic acid phosphatase 2B; *AGPAT6*  =  1-acylglycerol-3-phosphate O-acyltransferase 6; *SHROOM3*  =  shroom family member 3; *ALDH7A1*  =  aldehyde dehydrogenase 7 family, member A1; *AKR1B10*  =  aldo-keto reductase family 1, member B10; *DGKD*  =  diacylglycerol kinase; *GK5*  =  glycerol kinase 5; *GLA*  =  galactosidase, alpha; *MOGAT1*  =  monoacylglycerol O-acyltransferase 1; *AR*  =  androgen receptor; *LIPG*  =  endothelial lipase.

### DGEs results were confirmed by qRT-PCR

**Figure 7:**
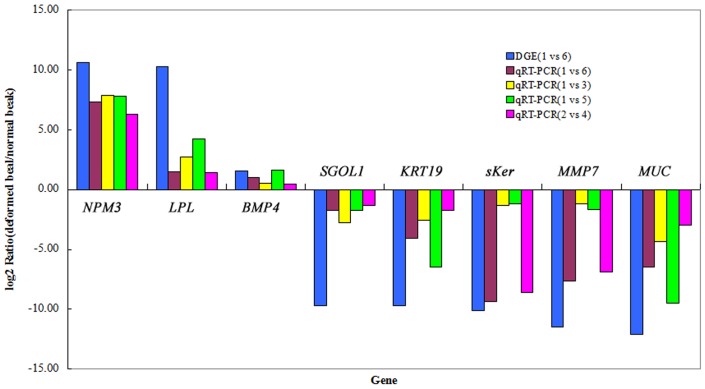
qRT-PCR of 8 transcripts for validating the DGE results. The horizontal axis identifies the 8 transcripts examined by qRT-PCR; the vertical axis shows the relative gene expression level in deformed (individuals 1 and 2) versus normal (individuals 3, 4, 5, and 6) beak tissues. The 4 pairs of individuals were all full sibs. The first bar shows the value obtained from DEG using 1 and 6. *NPM3*  =  nucleophosmin/nucleoplasmin 3; *LPL*  =  lipoprotein lipase; *BMP4*  =  bone morphogenetic protein 4; *SGOL1*  =  shugoshin-like 1; *KRT19*  =  Keratin 19; *sKer*  =  similar to Scale keratin; *MMP7*  =  matrix metalloproteinase 7; *MUC*  =  mucin protein.

To verify the foregoing DGE analysis, 8 genes *NPM3*, *LPL*, *BMP4*, *SGOL1*, *KRT19*, *sKer*, *MMP7* (matrix metalloproteinase 7), and *MUC* from the DEGs were examined by qRT-PCR using RNA from the beaks of 4 full-sib pairs, with and without the deformity phenotype. The results (Figure 7) showed good agreement with the DGE analysis pattern, indicating reliability of the latter.

## Discussion

The molecular genetic mechanism underlying beak deformity is likely to be very complex. Based on the DGE and bioinformatics analyses used in the present study, we identified the DEGs in the deformed and normal beaks, some of which were quite extreme, and found the enriched pathways of the DEGs. Some DEGs and pathways were selected as the likely candidate genes related to beak deformity.

### DEGs between the deformed and normal beaks

In the present study, 1,156 DEGs with more than 2-fold expression difference were identified. Of these, about a third were up-regulated and the remainder were down-regulated in the anomalous beak. Three genes were selected as promising candidate genes on the basis of their known functions as well as their differential ratios, identified here. The most profoundly up-regulated gene (log2-Ratio (deformed beak/normal beak)  = 10.91) was *LOC426217*, a hypothetical gene member of the keratin family [23]. Keratin is an important protein of the cytoskeleton playing a critical role in maintaining cell morphology [24]. It is also an intermediate filament protein that has essential functions in maintaining the structural integrity of epidermis and its appendages [25], presumably including the beak. Although, the key mechanistic role of *LOC426217* is not yet known, our DEGs results suggest its having a role in maintaining beak morphology. At the other extreme, *MUC* was the most down-regulated gene (log2-Ratio (deformed beak/normal beak)  = −12.11, i.e. >4000-fold). It is also a gene nearby many gene families [26], the expression product of which is mucin, a cohort of highly glycosylated proteins produced by epithelial tissues and implicated in bone formation [27]. Already shown by others to be a key gene in the development of beak morphology [13], [28], [29], [30], and shown here to be somewhat up-regulated (log2-Ratio (deformed beak/normal beak)  = 1.55), *BMP4* is included in our group of provisional candidate genes associated with beak deformity.

### GO and pathway enrichment analysis of DEGs

GO enrichment analysis showed that the DEGs were significantly enriched on the ontology of cellular component where they were mainly associated with intracellular, cell organs, and cytoskeleton categories. This indicated that the enriched DEGs play their roles mainly in these categories. It is worth mentioning that 7 DEGs including the most up-regulated gene *LOC426217* are members of the keratin family, located on GGA 25. As stated earlier, keratin is crucial for maintaining normal cell morphology and change of its structure results in dysmorphic cells [24]. Variation of keratin structure can lead to beak deformity [31]. The cytoskeleton is a complex of intracellular proteins that contribute to shape, support, and movement of cells [32]. The enrichment of keratin family gene DEGs in the cytoskeleton category may indicate their role in cell support. Their abnormal expression in the beak may therefore result in a deformed beak, although further functional study of these genes, especially that of *LOC426217*, is needed.

To shed more lights into the functional roles of DEGs responsible for the deformity beak, biological metabolic pathways were investigated by the enrichment analysis of DEGs. Pathway enrichment analysis showed that those involving biosynthesis of unsaturated fatty acids and glycerolipid metabolism were significantly enriched pathways of the DEGs exposed here. They could be target pathways in the development of beak deformity and the genes selected from the pathways above could be important candidate genes related to beak deformity. Four DEGs from the 2 pathways were selected, consisting of *ACAA1* from unsaturated fatty acid biosynthesis, and *LPL*, *ALDH7A1*, and *GLA* from glycerolipid metabolism. *ACAA1* is known to be related to the formation and development of teeth [33]. *LPL* encodes the extracellular lipase enabling acyltransfer between blood and tissue lipids. *ALDH7A1* functions in lipid peroxidation, and *GLA* is involved in glycerolipid hydrolysis. The relevance of these genes and their involved pathways to beak deformity may relate to the high content (up to 21%) of lipid in beak tissue [8] where it has a critical function as an extracellular matrix for the keratinized cells. Previous studies indicated that the disruption of retinol metabolism can cause abnormal development of the beak [34], [35] and influences the first branchial arch cartilages of chicken embryos [36]. Our results, however, did not show enrichment of the DEGs on this pathway but 4 DEGs identified here did relate to retinol acid metabolism, Retinol saturase (*RETSAT*), short chain dehydrogenase/reductase family 16C, member 5 (*SDR16C5*), WW domain containing oxidoreductase (*WWOX*), and Monoacylglycerol O-acyltransferase 1 (*MOGAT1*). They may be interesting candidate genes for beak deformity, worth further study.

The morphology of the mature beak reflects the sum of all proceeding developmental and growth processes, much of which remains poorly understood. There has been extensive investigation of normal chicken craniofacial development and some of the determining factors such as *BMPs*, *Shh*, and *FGFs*, have been documented [9], [29], [34]. For these reasons, exposing the anomalous transcriptome at 56 days of age cannot be expected to identify all causative genes, nor can the DEG analysis alone fully expose possible post-transcriptional defects underlying the deformity. This approach does, however, identify genes with atypical expression and potentially establishes a cohort of candidates for seeking gene variants and post-transcriptional modifiers. To increase the scope and power of future planned studies, a breeding population of Beijing-You chickens, from founders with deformed beaks, is being established.

## Conclusions

To the best of our knowledge, this is the first time the genes related to beak deformity have been identified using genome-wide gene expression analysis. The results suggested that 11 candidate genes are deserving of further study: *MUC*, *LOC426217*, *BMP4*, *ACAA1*, *LPL*, *ALDH7A1*, *GLA*, *RETSAT*, *SDR16C5*, *WWOX*, and *MOGAT1*. Several of the genes correspond to the cellular component GO category and KEGG analysis showed the importance of pathways of unsaturated fatty acid biosynthesis and glycerolipid metabolism. Subsequent studies including exploring, analysis of network, sequencing analysis, and functional verification will be done in these candidate genes, which could ultimately reveal the pinpoint causes of beak deformity and the underlying mechanisms of the disorder in chickens, as well as wild birds.

## Supporting Information

Figure S1 **The biosynthesis of unsaturated fatty acid pathway.** The up-regulated genes were marked with red color and down-regulated genes with green color.
(TIF)

Figure S2 **The glycerolipid metabolism pathway.** The up-regulated genes were marked with red color and down-regulated genes with green color.
(TIF)

Table S1 **The up-regulated genes with the (log2-Ratio (deformed beak/normal beak) ≥2).**
(DOC)

Table S2 **The down-regulated genes with the (log2-Ratio (deformed beak/normal beak) ≤−2).**
(DOC)
