# Supplementary figures and images for: Identification of Genes Related to Beak Deformity of Chickens Using Digital Gene Expression Profiling
Source: PLoS One. 2014 Sep 8;9(9):e107050. doi: 10.1371/journal.pone.0107050 (PMC4157856; doi:10.1371/journal.pone.0107050)

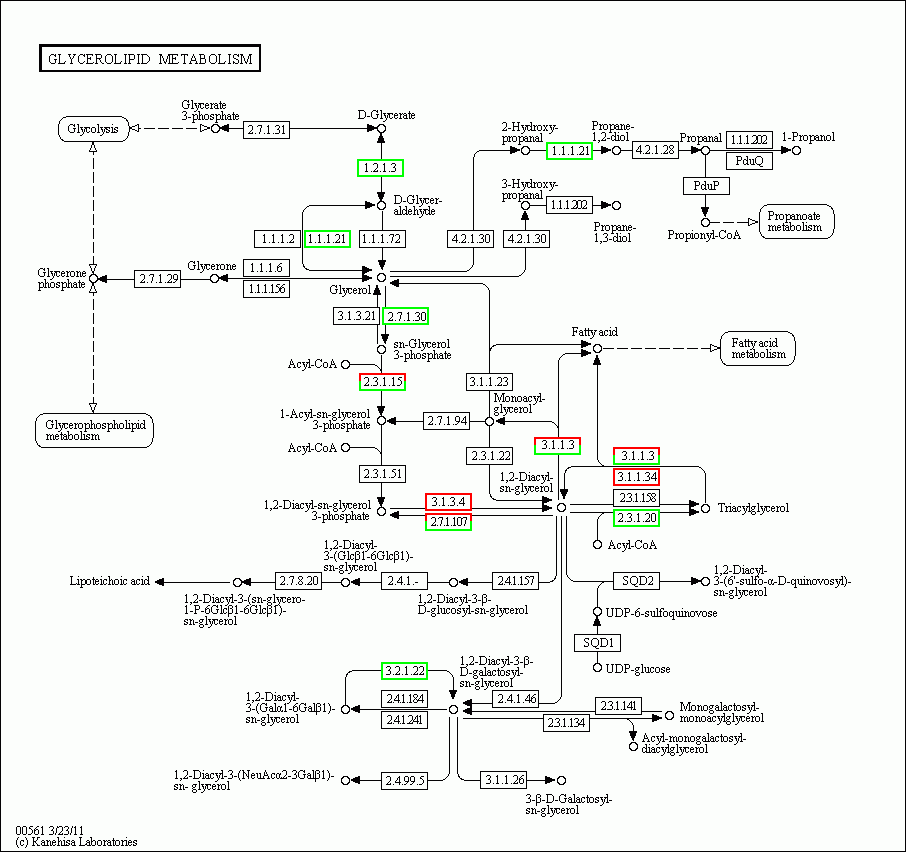

Supplement: Figure S1 — The biosynthesis of unsaturated fatty acid pathway. The up-regulated genes were marked with red color and down-regulated genes with green color. (TIF) [file pone.0107050.s001.tif]

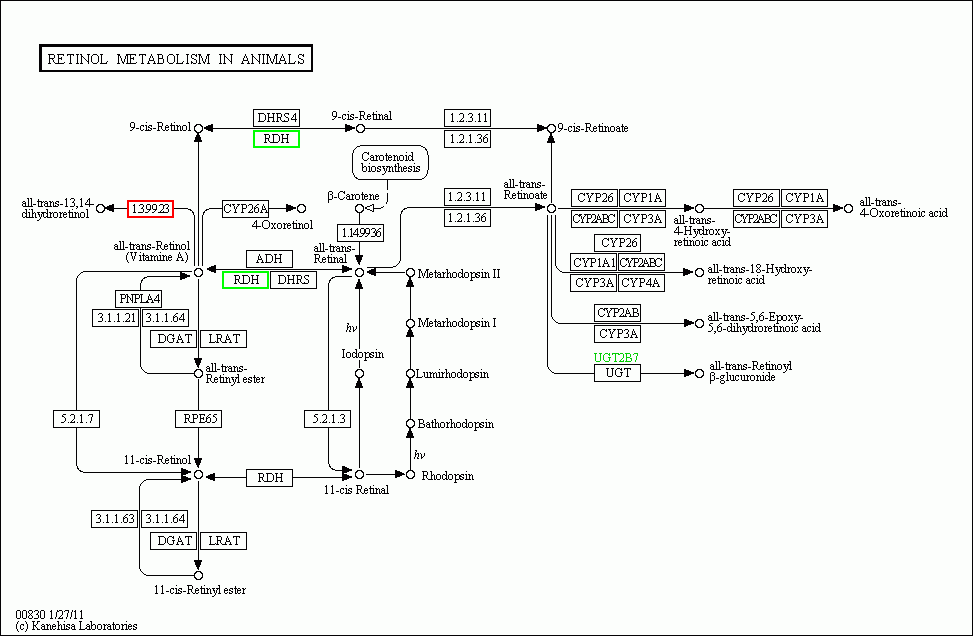

Supplement: Figure S2 — The glycerolipid metabolism pathway. The up-regulated genes were marked with red color and down-regulated genes with green color. (TIF) [file pone.0107050.s002.tif]
