# Supplementary material for: Identification of Genes Related to Beak Deformity of Chickens Using Digital Gene Expression Profiling
Source: PLoS One. 2014 Sep 8;9(9):e107050. doi: 10.1371/journal.pone.0107050 (PMC4157856; doi:10.1371/journal.pone.0107050)
Supplement: Table S1 — The up-regulated genes with the (log2-Ratio (deformed beak/normal beak) ≥2). (DOC) [file pone.0107050.s003.doc]

Supplemental Table S1. The up-regulated genes with the (log2-Ratio (deformed beak/normal beak) ≤ 2)

| Gene | Log2-Ratio |
| --- | --- |
| similar to keratin | 10.91 |
| nucleophosmin/nucleoplasmin 3 | 10.62 |
| lipoprotein lipase | 10.25 |
| retinol binding protein 7 cellular | 10.17 |
| nucleotide binding protein 2 (MinD homolog E. coli) | 10.00 |
| ADP-ribosylation factor-like 6 interacting protein 1 | 9.73 |
| musculin (activated B-cell factor-1) | 9.61 |
| ring finger protein 213 | 9.54 |
| THO complex 3 | 9.00 |
| benzodiazapine receptor (peripheral)-like 1 | 8.95 |
| growth arrest-specific 7 | 8.67 |
| patatin-like phospholipase domain containing 2 | 8.54 |
| nuclear receptor co-repressor 2 | 8.39 |
| interleukin 8 | 8.14 |
| fatty acid binding protein 4 adipocyte | 8.06 |
| v-ets erythroblastosis virus E26 oncogene homolog 2 (avian) | 8.06 |
| phosphatidic acid phosphatase type 2A | 7.95 |
| arylacetamide deacetylase-like 1 | 7.95 |
| hypothetical protein LOC768370 | 7.95 |
| retinol saturase (all-trans-retinol 1314-reductase) | 7.85 |
| telomeric repeat binding factor 2 interacting protein | 7.85 |
| four and a half LIM domains 3 | 7.73 |
| G protein-coupled receptor 133 | 7.73 |
| transmembrane protein 177 | 7.73 |
| transforming growth factor beta receptor II (70/80kDa) | 7.73 |
| forkhead box N1 | 4.92 |
| F-box and leucine-rich repeat protein 5 | 4.78 |
| hypothetical LOC425531 | 4.6 |
| prostaglandin-D synthase | 4.38 |
| prostaglandin E synthase 2 | 4.23 |
| glucagon receptor precursor | 4.17 |
| hypothetical LOC421245 | 3.91 |
| T-box 22 | 3.81 |
| eukaryotic translation initiation factor 4E binding protein 2 | 3.81 |
| gap junction protein gamma 1 45kDa | 3.58 |
| similar to Lysozyme g (14-beta-N-acetylmuramidase) (Goose-type lysozyme) | 3.42 |
| hypothetical protein LOC771882 | 3.35 |
| similar to pcmt1 protein | 3.31 |
| protein tyrosine phosphatase receptor type M | 3.28 |
| glutamine-fructose-6-phosphate transaminase 2 | 3.25 |
| thyroid hormone responsive (SPOT14 homolog rat) | 3.24 |
| apolipoprotein A-I | 3.06 |
| giant axonal neuropathy (gigaxonin) | 3.01 |
| Kruppel-like factor 15 | 2.97 |
| similar to complement C4-1 | 2.96 |
| collagen type VIII alpha 2 | 2.95 |
| tRNA methyltransferase 11 homolog (S. cerevisiae) | 2.92 |
| gap junction protein, beta 1, 32kDa (GJB1) | 2.89 |
| angiotensin II receptor type 2 | 2.87 |
| LIM and cysteine-rich domains 1 | 2.82 |
| arsenic (+3 oxidation state) methyltransferase | 2.81 |
| hypothetical protein LOC769518 | 2.81 |
| ring finger protein 103 | 2.75 |
| G protein-coupled receptor 124 | 2.74 |
| tumor necrosis factor alpha-induced protein 6 | 2.7 |
| KCNMB2 | 2.69 |
| tensin 1 | 2.67 |
| ATP-binding cassette sub-family B (MDR/TAP) member 1 | 2.64 |
| glutaminyl-peptide cyclotransferase (glutaminyl cyclase) | 2.64 |
| noggin 2 | 2.64 |
| hyaluronan synthase 2 | 2.59 |
| hydroxy-delta-5-steroid dehydrogenase 3 beta- and steroid delta-isomerase 1 | 2.59 |
| similar to ALDH7 | 2.58 |
| zinc finger DHHC-type containing 2 | 2.58 |
| neuritin 1 | 2.55 |
| similar to Wpkci; histidine triad nucleotide binding protein W | 2.54 |
| hydroxysteroid (11-beta) dehydrogenase 1-like | 2.47 |
| chromosome 1 open reading frame 77 | 2.45 |
| guanine nucleotide binding protein alpha inhibiting activity polypeptide 3 | 2.42 |
| ATP-binding cassette sub-family A (ABC1) member 8 | 2.41 |
| lysozyme G-like 2 | 2.39 |
| similar to Activated in blocked unfolded protein response protein 2 | 2.38 |
| cathepsin G | 2.38 |
| ecotropic viral integration site 5 | 2.37 |
| SNW domain containing 1 | 2.36 |
| CDP-diacylglycerol synthase (phosphatidate cytidylyltransferase) 2 | 2.35 |
| cell division cycle 2-like 5 (cholinesterase-related cell division controller) | 2.35 |
| scavenger receptor class A member 5 (putative) | 2.33 |
| potassium channel tetramerisation domain containing 10 | 2.31 |
| SERTA domain containing 2 | 2.31 |
| lipoma HMGIC fusion partner | 2.31 |
| COMM domain containing 10 | 2.28 |
| zinc finger ZZ-type with EF-hand domain 1 | 2.28 |
| serpin peptidase inhibitor clade B (ovalbumin) member 10 | 2.23 |
| milk fat globule-EGF factor 8 protein | 2.21 |
| hypothetical LOC424918 | 2.19 |
| microfibrillar associated protein 5 | 2.17 |
| cysteine-rich angiogenic inducer 61 | 2.15 |
| sirtuin (silent mating type information regulation 2 homolog) 1 (S. cerevisiae) | 2.13 |
| similar to keratin; keratin | 2.13 |
| transmembrane and tetratricopeptide repeat containing 1 | 2.12 |
| tripartite motif-containing 29 | 2.12 |
| epithelial membrane protein 1 | 2.09 |
| lactotransferrin | 2.08 |
| glycerol-3-phosphate dehydrogenase 1 (soluble) | 2.07 |
| phosphatidic acid phosphatase type 2B | 2.06 |
| chromosome 8 open reading frame 33 | 2.06 |
| transglutaminase 2 | 2.03 |
| sphingosine kinase 1 | 2.03 |
| secreted phosphoprotein 1 (SPP1) | 2.02 |
| peroxisome proliferator-activated receptor gamma | 2.01 |
| nuclear protein localization 4 homolog (S. cerevisiae) | 2.00 |
| similar to Scale keratin (S-ker) (sKer); keratin | 2.00 |
