# Supplementary material for: Identification of Genes Related to Beak Deformity of Chickens Using Digital Gene Expression Profiling
Source: PLoS One. 2014 Sep 8;9(9):e107050. doi: 10.1371/journal.pone.0107050 (PMC4157856; doi:10.1371/journal.pone.0107050)
Supplement: Table S2 — The down-regulated genes with the (log2-Ratio (deformed beak/normal beak) ≤−2). (DOC) [file pone.0107050.s004.doc]

Supplemental Table S2. The down-regulated genes with the (log2-Ratio (deformed beak/normal beak) ≤ -2)

| Gene | Log2-Ratio |
| --- | --- |
| transmembrane protease serine 2 | -2.00 |
| mannosidase alpha class 2B member 2 | -2.01 |
| metal response element binding transcription factor 2 | -2.02 |
| LAG1 homolog ceramide synthase 4 | -2.03 |
| tumor protein p53 binding protein 1 | -2.04 |
| non-SMC condensin II complex subunit G2 | -2.04 |
| peroxisomal biogenesis factor 6 | -2.04 |
| chromosome 1 open reading frame 91 | -2.05 |
| ADP-ribosylation factor guanine nucleotide-exchange factor 2 | -2.09 |
| acyl-Coenzyme A binding domain containing 5 | -2.10 |
| hypothetical protein LOC770534 | -2.10 |
| arginine-rich mutated in early stage tumors | -2.10 |
| similar to KIAA2026 protein | -2.10 |
| progestin and adipoQ receptor family member III | -2.10 |
| agrin | -2.12 |
| similar to keratin 10; cytokeratin 10 | -2.13 |
| acyl-CoA synthetase bubblegum family member 2 | -2.13 |
| similar to replication factor C large subunit | -2.14 |
| ectonucleoside triphosphate diphosphohydrolase 6 (putative function) | -2.14 |
| serpin peptidase inhibitor clade H (heat shock protein 47) member 1 | -2.14 |
| solute carrier family 12 (sodium/potassium/chloride transporters) member 2 | -2.16 |
| GTP cyclohydrolase I feedback regulator | -2.17 |
| SEC24 related gene family member D (S. cerevisiae) | -2.17 |
| annexin A6 | -2.17 |
| centromere protein H | -2.18 |
| transmembrane protein 116 | -2.19 |
| thymidylate synthetase | -2.19 |
| similar to LN1; phytanoyl-CoA 2-hydroxylase | -2.20 |
| chromosome 20 open reading frame 59 | -2.21 |
| crystallin alpha B | -2.22 |
| espin | -2.22 |
| asparagine-linked glycosylation 8 homolog | -2.23 |
| ADP-ribosylation factor | -2.23 |
| IKAROS family zinc finger 5 (Pegasus) | -2.24 |
| polo-like kinase 1 (Drosophila) | -2.25 |
| glycerophosphodiester phosphodiesterase 1 | -2.27 |
| dual specificity phosphatase 11 (RNA/RNP complex 1-interacting) | -2.27 |
| BUB1 budding uninhibited by benzimidazoles 1 homolog (yeast) | -2.27 |
| inosine triphosphatase (nucleoside triphosphate pyrophosphatase) | -2.27 |
| similar to Chromosome condensation protein G | -2.27 |
| LOC418413 | -2.27 |
| similar to hypothetical protein MGC11324 | -2.28 |
| hypothetical protein LOC769402 | -2.29 |
| tec protein tyrosine kinase | -2.31 |
| tetratricopeptide repeat domain 12 | -2.31 |
| hydroxysteroid dehydrogenase like 1 | -2.31 |
| mitochondrial ribosomal protein S16 | -2.32 |
| NmrA-like family domain containing 1 | -2.33 |
| regulator of G protein signalling 9 binding protein | -2.33 |
| thioredoxin domain containing 13 | -2.36 |
| 3-hydroxybutyrate dehydrogenase type 1 | -2.36 |
| maternal embryonic leucine zipper kinase | -2.37 |
| similar to collagen type XIV alpha 1 (undulin) | -2.37 |
| similar to class II histocompatibility antigen M beta chain 2 | -2.37 |
| myeloid cell leukemia sequence 1 (BCL2-related) | -2.39 |
| discs large homolog 7 (Drosophila) | -2.39 |
| insulin-like growth factor binding protein 2 36kDa | -2.40 |
| karyopherin alpha 2 (RAG cohort 1 importin alpha 1) | -2.41 |
| hypothetical LOC427084 | -2.41 |
| kinesin family member 20A | -2.41 |
| cytochrome P450 family 39 subfamily A polypeptide 1 | -2.43 |
| solute carrier family 5 (sodium/glucose cotransporter) member 1 | -2.45 |
| hypothetical LOC423006 | -2.45 |
| UDP-N-acetyl-alpha-D-galactosamine | -2.48 |
| centromere protein F, 350/400kDa (mitosin) (CENPF) | -2.48 |
| tenascin N | -2.51 |
| MYC induced nuclear antigen | -2.52 |
| GALNT12 | -2.53 |
| quiescence-specific protein | -2.53 |
| similar to ceramide kinase; ceramide kinase | -2.55 |
| lipopolysaccharide-induced TNF factor | -2.58 |
| similar to hypothetical protein MGC52498 | -2.58 |
| matrix metallopeptidase 2 | -2.61 |
| collagen type XXI alpha 1 | -2.61 |
| similar to ovomacroglobulin ovostatin | -2.63 |
| cell division cycle associated 3 | -2.68 |
| osteocrin | -2.69 |
| pleckstrin homology domain containing family G member 5 | -2.70 |
| telomeric repeat binding factor (NIMA-interacting) 1 | -2.72 |
| similar to hensin | -2.73 |
| hypothetical gene supported by CR385186 | -2.77 |
| mal T-cell differentiation protein | -2.77 |
| nucleoporin 133kDa | -2.77 |
| deiodinase, iodothyronine, type II (DIO2) | -2.78 |
| similar to RIKEN cDNA 2700049P18 | -2.80 |
| synuclein beta | -2.82 |
| tRNA splicing endonuclease 2 homolog (S. cerevisiae) | -2.82 |
| chromosome 6 open reading frame 192 | -2.82 |
| dishevelled dsh homolog 1 (Drosophila) | -2.85 |
| centrosomal protein 78kDa | -2.89 |
| mediator of RNA polymerase II transcription subunit 8 homolog | -2.89 |
| synaptosomal-associated protein 91kDa homolog (mouse) | -2.89 |
| reelin | -2.89 |
| hypothetical protein LOC776374; dihydroxyacetone kinase 2 homolog | -2.89 |
| structural maintenance of chromosomes 2 | -2.90 |
| endoplasmic reticulum metallopeptidase 1 | -2.90 |
| keratin 4 | -2.92 |
| ladinin 1 | -2.94 |
| similar to similar to aldose reductase | -3.01 |
| lipase endothelial | -3.01 |
| adaptor-related protein complex 1 sigma 3 subunit | -3.03 |
| topoisomerase (DNA) II alpha 170kDa | -3.04 |
| inturned planar cell polarity effector homolog (Drosophila) | -3.05 |
| cytoskeleton associated protein 2 | -3.08 |
| CNDP dipeptidase 2 (metallopeptidase M20 family) | -3.14 |
| solute carrier family 13 (sodium-dependent citrate transporter) member 5 | -3.15 |
| ubiquitin carboxyl-terminal esterase L1 | -3.15 |
| similar to antithrombin 1 B chain | -3.18 |
| hypothetical LOC418360 | -3.19 |
| alpha-2-macroglobulin | -3.21 |
| non-SMC condensin II complex subunit H2 | -3.28 |
| hypothetical LOC423119 | -3.37 |
| vitronectin | -3.38 |
| alpha-2-macroglobulin; similar to ovomacroglobulin ovostatin | -3.41 |
| coagulation factor VIII procoagulant component | -3.45 |
| cornulin | -3.49 |
| DEP domain containing 1B | -3.53 |
| xylosyltransferase I | -3.61 |
| claudin 22 | -3.67 |
| PDZ and LIM domain 7 (enigma) | -3.67 |
| polymeric immunoglobulin receptor | -3.72 |
| Gal 5 | -3.77 |
| diacylglycerol lipase beta | -3.77 |
| immunoglobulin J polypeptide | -3.90 |
| similar to epididymal protein | -4.05 |
| calcium channel voltage-dependent beta 4 subunit | -4.17 |
| HEG homolog 1 (zebrafish) | -4.24 |
| transglutaminase 4 (prostate) | -4.28 |
| lysozyme (renal amyloidosis) | -4.39 |
| interferon regulatory factor 4 | -4.43 |
| chitinase acidic | -4.54 |
| tetraspanin 1 | -5.99 |
| regenerating islet-derived family member 4 | -6.24 |
| keratin 75 | -7.49 |
| COX10 homolog cytochrome c oxidase assembly protein heme A | -7.79 |
| myosin light chain 3 alkali | -7.79 |
| uroplakin 1B | -7.79 |
| similar to potassium inwardly-rectifying channel J5 | -7.90 |
| kelch-like 4 (Drosophila) | -7.90 |
| c-ros oncogene 1 , receptor tyrosine kinase (ROS1) | -7.90 |
| golgi phosphoprotein 3-like | -8.00 |
| TRAF interacting protein | -8.00 |
| ATPase type 13A4 | -8.00 |
| similar to exocyst complex component 8 | -8.00 |
| similar to Scale keratin (S-ker) (*sKer*) | -8.09 |
| hypothetical LOC418426 | -8.09 |
| chromosome 17 open reading frame 38 | -8.18 |
| actin-related protein 10 homolog (S. cerevisiae) | -8.18 |
| similar to similar to Ataxin 2-binding protein 1; ataxin 2-binding protein 1 | -8.26 |
| SAM pointed domain containing ets transcription factor | -8.26 |
| tumor suppressor candidate 3 | -8.26 |
| hypothetical protein LOC769729; hypothetical LOC426064 | -8.34 |
| WD repeat domain 24 | -8.34 |
| X-ray repair complementing defective repair in Chinese hamster cells 2 | -8.61 |
| pleckstrin homology domain containing family F member 1 | -8.68 |
| similar to macrophage inflammatory protein-2 | -8.68 |
| DiGeorge syndrome critical region gene 14 | -9.09 |
| chromosome 3 open reading frame 38 | -9.09 |
| keratin 19 | -9.68 |
| shugoshin-like 1 (S. pombe) | -9.68 |
| similar to Scale keratin (S-ker) (*sKer*) | -10.09 |
| matrix metallopeptidase 7 (matrilysin uterine) | -11.45 |
| mucin protein | -12.11 |
